# Reply: Remarks on the BOADICEA model of genetic susceptibility to breast and ovarian Cancer Research UK

**DOI:** 10.1038/sj.bjc.6602488

**Published:** 2005-03-22

**Authors:** A C Antoniou, P D P Pharoah, A P Cunningham, D F Easton

**Affiliations:** 1 Cancer Research UK, Genetic Epidemiology Unit, Strangeways Research Laboratory, Department of Public Health and Primary Care, University of Cambridge CB1 4RN, UK; 2 Cancer Research UK, Human Cancer Genetics Group, Department of Oncology, University of Cambridge, CB14RN, UK

**Sir**,

The BOADICEA model of genetic susceptibility to breast and ovarian cancer was developed using complex segregation analysis of breast and ovarian cancer (Antoniou *et al*, 2002, 2004). We agree with van Asperen *et al* that as it stands, the model is not easy to use in clinical practice. However, the model is currently being implemented in web-based software that will provide a user-friendly tool for clinical geneticists and oncologists. We disagree however with the premise that the model is particularly hard to understand. The BOADICEA model incorporates the effects of BRCA1, BRCA2 and other genes. Although it has more risk parameters than some other models, it is conceptually quite similar to the Claus *et al* (1991) or BRCAPRO models (Parmigiani *et al*, 1998), or the model proposed by Jonker *et al* (2003). The major difference is the incorporation of a polygenic component to explain familial aggregation of breast cancer not attributable to BRCA1 and BRCA2.

van Asperen *et al* question the low breast cancer risk (13% by age 70) estimated for the index woman in Figure 3 of our paper. We agree that this estimate is probably anomalously low, due to imprecision in the BRCA1 and BRCA2 penetrance estimates we used. The average risk of breast cancer in BRCA1 mutation carriers in the first version of the BOADICEA model was estimated to be 35% by age 70 (Antoniou *et al*, 2002), which is much lower than the estimates used in the Jonker *et al* (2003). However, the BRCA1 and BRCA2 incidence rates used in the first version of BOADICEA were based on relatively small numbers of BRCA1 and BRCA2 mutation positive families (62 in total) and may therefore be imprecise.

To improve the risk prediction, we have recently refitted the BOADICEA model using additional data from two UK population-based studies of breast cancer (Peto *et al*, 1999; Lalloo *et al*, 2003) and data from the meta-analysis of the families of BRCA1/2 carriers identified through population-based studies of breast and ovarian cancer (Antoniou *et al*, 2003). The updated data set includes more than 500 BRCA1 and BRCA2 mutation positive families, and therefore the incidence rates are estimated more reliably (manuscript in preparation). In the updated version, the average risk of breast cancer in BRCA1 mutation carriers by age 70 varies between 50 and 59% depending on the year of birth. Applying the latest version of the model to the family in Figure 2 of Antoniou *et al* (2004), the 40-year-old woman is predicted to carry a BRCA1 mutation with probability 41% and a BRCA2 mutation with a probability 1% (very similar to the previous estimates). However, her predicted risk of developing breast cancer by age 70 is now higher, 28%, perhaps closer to the expectations of van Asperen and co-workers.

van Asperen *et al* question the fact that the presence of ovarian cancer in the family does not affect the breast risk. It is a feature common to BOADICEA and all the other risk prediction models, however, that the risks of breast and ovarian cancer in a family are assumed to be independent given the BRCA1 and BRCA2 genotypes. Thus, the presence of ovarian cancer in the family only affects the breast cancer risk in so far as it affects the BRCA1 and BRCA2 carrier probabilities.

We agree that the regression model of van Asperen *et al* (2004) can be more easily used in clinical practice. However, this model cannot deal with the complex family histories seen in genetic clinics. Moreover, the model was derived by linear regression of the independent variables on the predictions given the Jonker *et al* (2003) model. Therefore, the validity of the regression formula critically depends on an as yet (to our knowledge) unvalidated model. In any event, evaluation of the accuracy of any model in predicting the correct carrier and cancer risks should be based on validation studies in independent series and not on the basis of individual families.
